# Regulatory landscape of nanotechnology and nanoplastics from a global perspective

**DOI:** 10.1016/j.yrtph.2021.104885

**Published:** 2021-06

**Authors:** Jacqueline Allan, Susanne Belz, Arnd Hoeveler, Marta Hugas, Haruhiro Okuda, Anil Patri, Hubert Rauscher, Primal Silva, William Slikker, Birgit Sokull-Kluettgen, Weida Tong, Elke Anklam

**Affiliations:** a Joint Institute for Innovation Policy, Belgium; b European Commission, Joint Research Centre (JRC), Italy; c European Food Safety Authority (EFSA), Italy; d National Institute of Health Sciences (NIHS), Japan; e National Center for Toxicological Research (NCTR), Food and Drug Administration (FDA), USA; f Canadian Food Inspection Agency, Canada; g European Commission, Joint Research Centre (JRC), Belgium

**Keywords:** Nanotechnology, Nanomaterials, Nanoplastics, Harmonisation, Regulatory science, Standards, GSRS

## Abstract

Nanotechnology and more particularly nanotechnology-based products and materials have provided a huge potential for novel solutions to many of the current challenges society is facing. However, nanotechnology is also an area of product innovation that is sometimes developing faster than regulatory frameworks. This is due to the high complexity of some nanomaterials, the lack of a globally harmonised regulatory definition and the different scopes of regulation at a global level. Research organisations and regulatory bodies have spent many efforts in the last two decades to cope with these challenges. Although there has been a significant advancement related to analytical approaches for labelling purposes as well as to the development of suitable test guidelines for nanomaterials and their safety assessment, there is a still a need for greater global collaboration and consensus in the regulatory field. Furthermore, with growing societal concerns on plastic litter and tiny debris produced by degradation of littered plastic objects, the impact of micro- and nanoplastics on humans and the environment is an emerging issue. Despite increasing research and initial regulatory discussions on micro- and nanoplastics, there are still knowledge gaps and thus an urgent need for action. As nanoplastics can be classified as a specific type of incidental nanomaterials, current and future scientific investigations should take into account the existing profound knowledge on nanotechnology/nanomaterials when discussing issues around nanoplastics.

This review was conceived at the 2019 Global Summit on Regulatory Sciences that took place in Stresa, Italy, on 24–26 September 2019 (GSRS 2019) and which was co-organised by the Global Coalition for Regulatory Science Research (GCRSR) and the European Commission's (EC) Joint Research Centre (JRC). The GCRSR consists of regulatory bodies from various countries around the globe including EU bodies. The 2019 Global Summit provided an excellent platform to exchange the latest information on activities carried out by regulatory bodies with a focus on the application of nanotechnology in the agriculture/food sector, on nanoplastics and on nanomedicines, including taking stock and promoting further collaboration. Recently, the topic of micro- and nanoplastics has become a new focus of the GCRSR. Besides discussing the challenges and needs, some future directions on how new tools and methodologies can improve the regulatory science were elaborated by summarising a significant portion of discussions during the summit. It has been revealed that there are still some uncertainties and knowledge gaps with regard to physicochemical properties, environmental behaviour and toxicological effects, especially as testing described in the dossiers is often done early in the product development process, and the material in the final product may behave differently. The harmonisation of methodologies for quantification and risk assessment of nanomaterials and micro/nanoplastics, the documentation of regulatory science studies and the need for sharing databases were highlighted as important aspects to look at.

## Highlights

• Overview on current regulatory framework of various regulatory bodies on nanotechnology and nanoplastics.
• Main regulatory challenges are the lack of global standardisation of nomenclature, test methods or characterisation.
• Research on micro-/nanoplastics to be intensified taking into account results available in the area of nanomaterials.
• Urgent need for standards (documentary, materials, methods) especially for micro-/nanoplastics.
• Urgent need to understand environmental contamination, human exposure and possible risks from small plastic particles.

## 1 Introduction

Nanotechnology is an area of product innovation that is developing faster than regulatory frameworks. It does not present a one-size-fits-all situation; there are many possible variants and associated characteristics of any single type of material. Since the 2000s, the development of nanotechnology has led to an enormous investment in nanomaterials production. The market for nanomaterials may reach up to USD 100 billion by 2025 (Savolainen et al., 2013; Research and Markets, 2020; Future Markets, 2019). Nanomaterials offer a wide range of functionalities due to their specific physical and chemical properties, which promote their use to meet the needs of a wide range of applications. They offer new avenues in diagnostics and therapeutics in healthcare, for example, and open up the opportunity to address previously unmet medical needs (Gajanan and Tijare, 2018; McNamara and Tofail, 2017) with a gradual increase in medical products that utilise nanomaterials. Nanotechnology also finds applications across food and agriculture, consumer products, transport and logistics, energy and environmental sectors. The number and variety of new and modified nanomaterials that are being developed and the range of applications has significantly increased over the last ten years (some examples in: Patra et al., 2018; Urmann et al., 2017). It is therefore of prime importance that regulatory science can keep pace with new developments. Safety assessment of nanomaterials is an important element in this respect. The ‘safe-by-design’ concept is valuable for the development of safe nanomaterials (Kraegeloh et al., 2018). Over the last two decades, scientists and regulatory bodies worldwide have developed guidelines for toxicological assessments and analytical methods to implement definitions and other regulatory requirements, such as labelling. However, the growing number of the different types of novel materials with increased complexity further complicates the challenges, merging nanotechnologies with other emerging technologies, which are incorporated into novel products.

Nanomaterials in general are often purposely made due to their unique properties but can also be unintentionally present in products, e.g. in powders due to the distribution of particle size. A specific type of nanomaterials, nanoplastics, are mainly encountered as degradation products of plastic materials. Although the benefits of plastics for society are unquestionable, there is an urgent need to better manage their value chain. In addition to growing concerns about plastic litter, tiny plastic fragments have become another major concern as they have been ubiquitously detected and can come into contact with the human body through exposure from air, water and food. Such plastic fragments are called nano- and microplastics as their external size range from nanometers up to well beyond the micrometer scale. It has become increasingly apparent that a broad and systemic approach is required to achieve sustainable actions and solutions along the entire plastic supply chain, with an urgent global need for the monitoring of the environment and food (Alexy et al., 2020). The composition of micro- and nanoplastics is heterogeneous as they degrade from a vast number of different plastics containing a wide number of additives and other substances. Despite an increasing number of research projects, there is a lack of suitable and validated analytical methods for sampling, identification and quantification of micro- and nanoplastics, as well as a lack of hazard and fate data, which would allow for their risk assessment. However, there is an overlap related to the challenges for risk assessment of nanomaterials in general and the more specific field of nanoplastics. It is therefore desirable to bring together the scientific communities related to these fields. This was one aim of the 2019 annual conference of Global Summit on Regulatory Science (GSRS).

The membership of the Global Coalition for Regulatory Science Research (GCRSR) – established in 2013 under the leadership of the US Food and Drug Administration (FDA) – is comprised of regulatory bodies from ten countries including the European Union (EU). The aim of this global partnership is to improve regulatory science research on the safety and efficacy of consumer products, including food and drugs. A focus is on emerging technologies and big data science through facilitation and promotion of the development of advanced regulatory science that is directly applicable to public health. Since 2013, GCRSR has hosted annual GSRS conferences as a platform for improved communication among the international regulators (Healy et al., 2016; Pettit et al., 2016; Slikker et al., 2018; Tong et al., 2015: Thakkar et al., 2020). The 2019 GSRS was the 9th consecutive annual summit and was held September 25–26 in Stresa (Italy) co-organised by the GCRSR and one of its EU members, the JRC.

The focus of this summit was on topics in nanotechnology related to advances in standards, medical devices/drugs and food, the safety assessment of nanomaterials and the emerging pollutant nanoplastics. It provided a platform for regulators, policy makers, and scientists to exchange views on innovative technologies, methods and regulatory assessments, as well as harmonising strategies via global collaboration.

**Fig. 1:**
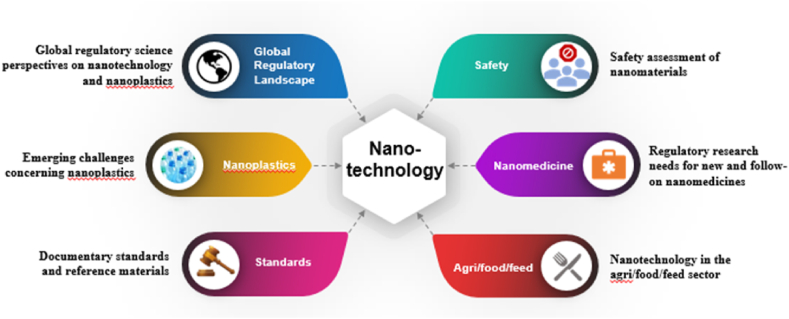
Topics discussed at GSRS2019.

The 2019 GSRS attracted around 200 participants from more than 30 countries representing wide geographical areas, including the USA, Canada, Argentina, Chile, China, Japan, Singapore, Korea, India, Australia, New Zealand, Egypt and the European Union. Six major topics were discussed in plenary and parallel sessions of GSRS2019 as depicted in Fig. 1.

The 2019 summit concluded with a discussion focussed on what actions need to be taken and accomplished in these areas and identified research gaps and/or priorities in regulatory science. A focus was on the establishment of platforms, mechanisms, communities of practice, and networks to facilitate effective collaborations between international partners.

Additionally, the JRC offered training opportunities and laboratory visits at its Ispra site (Italy). A hands-on training prior to the summit (September 24) on determining size distributions of nanomaterials was provided to 28 participants from 12 countries. Participants had the opportunity to deepen their knowledge on nanoparticle size distributions, to apply specific measurement techniques and to discover the capabilities of the NanoDefiner e-tool, a decision support framework for the characterisation of potential nanomaterials developed in the European Union NanoDefine project (Mech et al., 2020a; Brüngel et al., 2019). Furthermore, 29 participants of the 2019 GSRS attended a short course on the use of reference materials including the estimation of measurement uncertainty organised by the JRC and another 40 attendees of 2019 GSRS visited several laboratories at the JRC-Ispra site on September 27. The summit provided also the opportunity for back-to-back additional satellite meetings such as the Nanomedicines Working Group Meeting of the International Pharmaceutical Regulators Programme (IPRP), the Annual Meeting of the European Food Safety Authority (EFSA) Nano Network, and a workshop related to standards for regulatory science of the ASTM International Committee on Nanotechnology.

This paper summarises the key topics discussed in GSRS 2019, including the regulatory framework of various regulatory bodies and their comparison and the conclusions on the way forward as to how to tackle the emerging challenges related to nanotechnology and nanoplastics.

## 2 Considerations on definitions, terminologies, and classifications

### 2.1 Nanomaterials

For many years now, nanotechnology and more particularly nanotechnology-based products and materials have demonstrated a huge potential for novel solutions to many of the current challenges society is facing. Manufactured nanomaterials are key components of innovative products, and many consumer products (including food) containing nanomaterials can be currently found on the global market. In order to gain acceptance, e.g. by consumers, of these new products, it is important that they are safe for humans and the environment. It is therefore of utmost importance that regulators keep pace with the rapid developments as some nanomaterials might have a higher hazard potential than others. For comparable safety assessments – following the concept of ‘safe-by-design’ – it is important to use harmonised approaches (see also Chapter 5). This requires the availability of appropriate analytical methods for the detection and determination of nanomaterials in a variety of matrices (e.g. environment and consumer goods) for exposure assessment, but also appropriate toxicological methods for hazard assessments. All stakeholders, including scientists from industry and regulatory bodies, need to have a deep and common understanding of the nature of the nanomaterial investigated, i.e. a clear definition of what is regulated and assessed.

However, due to the high complexity of nanomaterials and the different scopes of regulations, a uniform definition across all regulatory areas is very challenging to achieve, therefore hampering the uniform identification and quantification of such materials. Ideally, a regulatory definition should be comprehensive to include those nanomaterials that would pose risks, but on the other hand should not be overly broad so that the implementation is possible. A too broad definition is likely to lead to an information overload for regulators and to high regulatory compliance costs for all stakeholders (Dana, 2010). To-date, several definitions have been elaborated by the various stakeholders, being often inconsistent in their elements and scope. The EC Recommendation on the Definition of Nanomaterial (EC Nanomaterial Definition) was adopted in 2011, but is not legally binding (European Commission, 2011). This definition is horizontal, i.e., applicable across all regulatory fields, and is, in general, in line with other definitions worldwide, e.g. the one of the International Organization for Standardization (ISO) (ISO, 2015). It is based on the only feature that is common to all nanomaterials, which is the nanoscale external particle dimensions (1 nm–100 nm), and it covers only particulate solid materials (Rauscher et al., 2019a). It is important to note that obviously this definition does not differentiate between hazardous and non-hazardous materials. The regulatory and advisory definitions of the term ‘nanomaterial’ were reviewed together with a comparative assessment (Boverhof et al., 2015), concluding that the definitions are not in alignment. More recently, an assessment on the legal and practical challenges on classifying nanomaterials according to regulatory definitions was performed (Miernicki et al., 2019). The European Commission has undertaken a very considerable effort to provide clarifications of the key concepts and terms used in the EC Nanomaterial Definition (Rauscher et al., 2019a; Mech et al., 2020b) resulting also in a number of European research projects (with participation of the JRC), and elaborating guidance and methods for sector-specific legislation. The JRC also has provided a scientific-technical evaluation of options to clarify the definition and to facilitate its implementation (Rauscher et al., 2015).

To-date, the EC Nanomaterial Definition has been integrated, wholly or partly, into several pieces of European legislation addressing, e.g., biocides, chemicals and medical devices whereas the regulations on cosmetic products and novel foods feature their own nanomaterial definitions, which were introduced before the EC Nanomaterial Definition was published. Table 1 describes in more detail some of the definitions or criteria for nanomaterials as they were presented and discussed in GSRS19.

**Table 1:** Some definitions, descriptions and criteria for nanomaterials as discussed in GSRS19.

Description	Definition or criteria	Source
**EC** recommended definition of nanomaterial	Nanomaterial is a natural, incidental or manufactured material containing particles, in an unbound state or as an aggregate or as an agglomerate and where, for 50% or more of the particles in the number size distribution, one or more external dimensions is in the size range 1 nm–100 nm. In specific cases and where warranted by concerns for the environment, health, safety or competitiveness the number size distribution threshold of 50% may be replaced by a threshold between 1 and 50%. By derogation […] fullerenes, graphene flakes and single wall carbon nanotubes with one or more external dimensions below 1 nm should be considered as nanomaterials.	European Union (2011): 2011/696/EU
Guidance for Industry Considering Whether an **FDA**-Regulated Product Involves the Application of Nanotechnology	FDA will ask: 1.Whether a material or end product is engineered to have at least one external dimension, or an internal or surface structure, in the nanoscale range (approximately 1 nm–100 nm); 2.Whether a material or end product is engineered to exhibit properties or phenomena, including physical or chemical properties or biological effects, that are attributable to its dimension(s), even if these dimensions fall outside the nanoscale range, up to 1 μm (1000 nm).6	FDA (2014): http://www.fda.gov/Regulatory Information/Guidances/ucm257698.htm Docket number (FDA-2010-D-0530)
**US-EPA**	…chemical substances that are solids at 25 °C and atmospheric pressure and that are manufactured or processed in a form where the primary particles, aggregates, or agglomerates are in the size range of 1–100 nm (nm) and exhibit unique and novel characteristics or properties because of their size. The proposed rule would apply to chemical substances containing primary particles, aggregates, or agglomerates in the size range of 1–100 nm in at least one dimension.	Federal Register/Vol. 82, No. 8, 3641 (2017)
**ISO** definition of nanomaterial	Nanomaterial: material with any external dimension in the nanoscale or having an internal structure or surface structure in the nanoscale	ISO/TR 18401:2017(en)
**REACH** definition of nanoform	On the basis of the Commission Recommendation of October 18, 2011 on the definition of nanomaterial, a nanoform is a form of a natural or manufactured substance containing particles, in an unbound state or as an aggregate or as an agglomerate and where, for 50% or more of the particles in the number size distribution, one or more external dimensions is in the size range 1 nm–100 nm, including also by derogation fullerenes, graphene flakes and single wall carbon nanotubes with one or more external dimensions below 1 nm.	COMMISSION REGULATION (EU) 2018/1881 (amending REACH)

Concerning the term ‘Nanobiotechnology’ or ‘Bionanotechnology’ – an important scientific discipline for the application of nanomaterials in the life sciences sector and for risk assessment - there is no clear definition, but there is a high pace of scientific developments and a convergence of technologies. The term ‘bionano’ would refer to biological molecules that are used to design and develop complex structures at the nanoscale for a specific functionality (e.g. the so-called DNA-origami, RNA-origami and design proteins structures) in combination with nanomaterials (Puchner et al., 2014). DNA-origami is a self-assembly of DNA molecules, usually a long DNA-strand to which smaller DNA-strands attach at pre-determined sites to form the desired 2-D or 3-D structure and size (typically between 25 nm and 15 nm) onto which's surface molecules and nanomaterials can be coupled. Potential applications include tailored encapsulation structures (to-date mostly for cancer medication but also with agriculture/food/feed potential (see more in Section 5)), molecular tools and nano-reactors for the fabrication of nanomaterials, building bio-mimicking systems, sensors and nano-devices/robots. There is also a high potential to use DNA for data storage (Wang et al., 2017). RNA-origami is similar to, albeit lagging behind, DNA-origami. The former has potential to be used in larger quantities and offers additional possibilities to link to substances. Protein-origami would enable larger 3-D structures compared to DNA- and RNA-origami (Oomen, 2019). Dealing with these complex and advanced systems is difficult and brings new challenges.

### 2.2 Nanoplastics

Plastic debris is mainly formed in the environment by fragmentation of larger objects consisting of synthetic or modified natural polymers as an essential ingredient and which does not fulfil an intended function. Plastic debris can be further categorised according to size (including microplastics, sub-micron and nanoplastics), shape, colour and origin (Hartmann et al., 2019; Hartmann, 2019). When the fragmentation of small plastic particles continues, and the external dimensions of the plastic particles fall below a certain size (1000 nm or 100 nm, depending on the definition used, see Table 1), microplastics become nanoplastics, which can be regarded as a specific type of nanomaterial.

Primary microplastics are intentionally manufactured materials such as pellets, powders and scrubbers, while secondary microplastics result from breakdown of plastic material arriving for example to the marine environment (e.g. from fishing gears, cages, buoys, boats and packaging) or from in-use degradation (e.g. textile fibres and tyre abrasion). It is not yet clear how much microplastics tend to adsorb contaminants that are present in its surroundings, e.g. marine water, or attract living (micro-)organisms (marine invertebrates, bacteria, fungi, viruses) that may use them even as a substrate. Such microplastics can enter the food chain and may be ingested by humans through the consumption of fish and seafood, particularly those eaten completely, i.e. without the removal of their digestive tract (Alexy et al., 2020). In this context, it should be stressed that the number of primary plastic particles compared to secondary ones is less relevant.

Although a number of scientific organisations and regulatory bodies worldwide are increasingly investigating the fate of plastics and monitoring the occurrence of microplastics (less work has been done on nanoplastics to-date due to the manifold challenges, as discussed in Section 6), there is no formal or harmonised definition for these small plastic particles. An overview on the currently used definitions for microplastics (and nanoplastics) proposed by some selected organisations is given in Table 2.

**Table 2:** *Overview on some currently discussed definitions of micro- and nanoplastics*.

Organisation	Definition of Microplastics (MPs) and Nanoplastics (NPs)	Source
European Food Safety Authority (EFSA)	*MPs*: Heterogeneous mixture of differently shaped materials referred to as fragments, fibres, spheroids, granules, pellets, flakes or beads, in the range of 0.1–5000 μm (100 nm–5 mm) *NPs*: Material with any external dimension in the nanoscale or having internal structure or surface structure in the nanoscale (0.001–0.1 μm = 1–100 nm)	European Food Safety Authority EFSA (2016)
European Chemicals Agency (ECHA)	*MPs*: Consisting of solid polymer-containing particles, to which additives or other substances may have been added, and where ≥ 1% w/w of particles have (i) all dimensions 1 nm ≤ x ≤ 5 mm, or (ii), for fibres, a length of 3 nm ≤ x ≤ 15 mm and length to diameter ratio of >3	ECHA (2019) EC-SAM (2019)
International Standardization Organization (ISO)	*MPs*: Solid plastic particles insoluble in water with any dimension between 1 mm and 5 mm (large MP)/1 μm and 1000 μm (MP) *NPs:* plastic particles smaller 1 μm	ISO (2020)
United Nations (UN)	*MPs*: Particles in the size range 1 nm to <5 mm.	UN-GESAMP (2015)
European Academies	*MPs and NPs:* Tiny plastic particles of mixed shapes and sizes below 5 mm.	SAPEA (2019)
US National Oceanic and Atmospheric Administration	*MPs*: Any type of plastic fragment that is less than 5 mm in length	US NOAA (2009)

As it can be seen from Table 1 as well as from the scientific literature, there is currently a general agreement (with some exceptions, e.g. ISO) that microplastics include particles and fibres of plastic measuring less than 5 mm, while the definition of nanoplastics is less well established. The term *nanoplastics* may be linked to the more general term *nanomaterial*, by considering not only the external particle dimensions as criterion but also the chemical composition of the particles. As the term nanomaterial is well established – at least in EU legislation -, it would be helpful to base a definition of nanoplastics on the one of nanomaterial. Following this logic, nanoplastics would be a nanomaterial that fulfils the EC Recommendation for a definition of nanomaterial (.European Commission, 2011) *and* contains synthetic or semi-synthetic polymer(s). It is however an open question how large the fraction of polymer should be so that the material could qualify, as a whole, as nanoplastics. A proposal made by the European Chemicals Agency (ECHA) in 2019 for a microplastics definition shows that such a decision is far from trivial (ECHA, 2019). If intentionally added nanoplastics is considered a specific type of nanomaterial, the legal provisions for nanomaterials shall also apply to nanoplastics. An analogous approach can be conceived also for other jurisdictions worldwide, which use the term nanomaterial, albeit without formal definition.

To conclude, as for nanomaterials, an appropriate and harmonised (and implementable) definition is important for the purposes of reliably monitoring these small plastic particles in the environment, food and consumer products as well as for their safety assessment. (Frias and Nash, 2019; Gigault et al., 2018; Hartmann et al., 2019).

## 3 Global regulatory landscape on nanomaterials and nanoplastics

As mentioned above, safety assessment is an essential part of the process of product development and a prerequisite for their release to the market. Therefore, it is important to consider these elements as early as possible and continuously in the value chain. Nanotechnology is an area of product innovation to, e.g., enhance materials’ properties, reduce consumption of materials, alleviate waste, and support the reduction of emissions to the environment. Sharing of research results and research facilities is strengthening the science base for regulation of nanomaterials and nanotechnology-based products (Devasahayam, 2017, 2019; Hodge et al., 2009).

Although the invention of plastics can be seen as a real success in satisfying the needs of modern society, some of its adverse impacts have somewhat been neglected, hence leading to threats to the environment and therefore also posing a societal challenge. Globally, only about 15% of plastic waste is collected for recycling while the majority is ending up in a landfill or the environment, e.g. in the oceans. Therefore, there is a pressing need for regulatory measures, including those aiming at the issues associated with microplastics, greater transparency on the use of additives in plastics, and the improvement of recyclability (Alexy et al., 2020). In this respect and as a good start, the EU has recently adopted provisions to reduce the use of single-use plastic products which represent the largest share of all marine litter (Addamo et al., 2018) (European Commission COM, 2018). However, more efforts are necessary at a global level such as a review of the waste legislation worldwide, the implementation of an efficient waste management system, possible restrictions of primary, intentionally added microplastics; and appropriate labelling measures to raise awareness among consumers to improve the quality of waste collections. Policy makers need to reach out to industries by pushing for innovation and facilitating regulatory acceptance of new materials. To-date, robust data to understand the exposure of humans and the environment to micro- and nanoplastics are scarce and consequently the risks are still unknown, making this topic important in the area of regulatory science.

In the following section, information (not comprehensive) from the GCRSR Members and speakers of the 2019 GSRS is given on the regulatory landscape and future challenges of nanotechnology and nanoplastics.

### 3.1 European Union

For the EU an increasingly important approach to nanosafety is the concept of *safe-by-design: creating nanomaterials of tomorrow*. Products based on nanomaterials or on nanotechnology need to be safe for use across the full lifecycle, from production to waste, or recycling and reuse. Safe-by-design is an established general concept in industrial innovation, and it was initially formulated for nanomaterials in the EU flagship project NANoREG (Gottardo et al., 2017). The safe-by-design approach should be a means to drive innovation forward. Support mechanisms include digital innovation hubs and open innovation test beds. With research organisation at the core, Digital Innovation Hubs (European Commission, 2017) aim to be one-stop-shops for companies – especially SMEs, start-ups and mid-caps (companies with a market value between $2 and $10 billion) – to access technology including testing instruments, receive financial advice and make use of market intelligence and networking opportunities. Open innovation test beds (Horizon 2020, 2020a) offer access to the physical facilities, capabilities and services needed for the development, testing and upscaling of advanced materials in industrial environments (Lima da Cunha, 2019).

The European Chemicals Agency (ECHA), an independent European executive agency funded by the European Union, addresses the safety assessment of chemicals including manufactured nanomaterials regulated under the European Chemical legislation REACH EC 1907/2006 (European Commission, 2006). The REACH Regulation lays down the system within the European Union for the registration, evaluation, authorisation and restriction of chemicals. The cornerstones are the registration of chemicals based on the information provided by companies, and the evaluation of those chemicals by the EU Member States. However, REACH is just part of the EU legislation addressing nanomaterials, as they may also fall under pieces of legislation on occupational safety, cosmetics, food packaging, biocides, food and feed. In 2018, the amendment of REACH introduced nano-specific information requirements and new provisions for their chemical safety assessment and downstream user obligations (ECHA, 2020). To date, 37 substance registration dossiers contain information on nanoforms. There is no transition phase for the implementation of these new requirements, even though fully harmonised or standardised test methods may not be available yet. Accordingly, the development of tests methods became a matter of highest importance. In addition to its regulatory role, ECHA hosts the European Union Observatory for Nanomaterials that aims to provide objective and reliable information to the public and others on innovation and safety aspects of nanomaterials on the EU market (Sumrein, 2019; ECHA, 2020).

The European Food Safety Authority (EFSA), another independent European executive agency, assesses risks associated with food and the feed chain (food and feed safety). This includes nutrition and health claims, biological hazards, animal health and welfare, contaminants, feed and food additives, plant protection and plant health. EFSA provides fit-for-purpose scientific advice to inform European policy makers and support the regulation and their implementation for human, animals and plant health. It also uses environmental risk assessment to explore the possible impact of the food chain on the biodiversity of plant and animal habitats. To address the use of nanotechnologies and nanomaterials, EFSA established a scientific network for risk assessment of the use of nanotechnologies in food and feed (EFSA, 2020) to facilitate the exchange of information between EU Member States and to prioritise risk assessment activities. Furthermore, it has a specific working group on nanotechnologies in food and feed, and has published a Guidance on risk assessment of the application of nanoscience and nanotechnologies in the food and feed chain. The regulatory aspects of nanotechnology in the agriculture/feed/food sector in EU and non-EU countries were reviewed by Amenta and co-authors in 2015 (Amenta et al., 2015).

The European Medicines Agency (EMA) is another agency of the European Union. It operates a working definition of nanomedicines as medicinal products that are purposely designed systems for clinical applications and have at least one component at the nano-scale, leading to definable specific proprieties and characterises providing clinical advantages, the nanotechnology offering specific benefits in, for example, dosage, drug targeting or reduced toxicity (Perez de la Ossa and Bremer-Hoffmann, 2019).

The European Commission's Green Deal (European Commission, 2019) is an ambitious roadmap which aims for a climate-neutral, zero-pollution, sustainable, circular and inclusive economy. It drives the “New Industrial Strategy for Europe” (European Commission, 2020) and the upcoming new EU Chemicals Strategy for Sustainability. The European Green Deal is an integral part of the EC's strategy to implement the United Nation's 2030 Agenda for Sustainable development (United Nations, 2015). The Green Deal specifies that the European Commission will follow up on the 2018 plastics strategy (European Commission, 2018), focussing, among other issues, on measures to tackle intentionally added microplastics and unintentional releases of plastics, for example from textiles and tires, and the EC will develop a regulatory framework for biodegradable and bio-based plastics. The EC has already implemented measures on single use plastics (see below) and will propose further measures to address pollution from urban runoff and from new or particularly harmful sources of pollution such as microplastics and chemicals. An action on plastics was already identified as a priority in the Circular Economy Action Plan (European Commission, 2015), to help European businesses and consumers to use resources in a more sustainable way. The European Strategy for Plastics in a Circular Economy was adopted on January 2018 to transform the way plastic products are designed, used, produced and recycled in the EU. Among other measures, it proposes better design of plastic products, higher plastic waste recycling rates, more and better quality recyclates to boost the market for recycled plastics. The European Commission has already started the process to restrict the use of intentionally added microplastics. Responding to a request by the EC, ECHA prepared a restriction proposal for microplastic particles that are intentionally added to mixtures used by consumers or professionals. If adopted, the restriction could reduce the amount of microplastics released to the environment in the EU by about 400 thousand tonnes over 20 years. The increasing amount of microplastics from fragmentation of larger pieces of plastic waste, but also entering the environment directly, is recognised as a key challenge in the plastics strategy. Although the EU Directive 2019/904 (European Union, 2019) is in the first place a measure to reduce the steady increase in plastic waste generation and the leakage of plastic waste into the environment, it also helps as a consequence reducing the amount of microplastics coming from fragmentation. According to the plastics strategy, a regulatory framework for plastics with biodegradable properties will be established that also should help to slow down the accumulation of micro- and nanoplastics in the environment.

### 3.2 United States of America

The US-FDA defines regulatory science as ‘the science of devolving new tools, standards, and approaches to assess the safety, efficacy, quality, and performance of all FDA-regulated products’ (Goering, 2019). The FDA's approach to the regulation of nanotechnology is to accept that the promise, risk and uncertainty accompany all emerging technologies. This results in the decision not to introduce new and specific regulations just for nanomaterials, with the assumption that the existing framework is sufficient to regulate nanomaterials and products containing nanomaterials. In this respect, horizon scanning and internal reviews of product submissions are key components in the FDA approach.

Nanotechnology core facilities provide laboratory-testing capacity at FDA, enabling the development of testing methods and standards related to the safety assessment of nanomaterials or of products containing nanomaterials or based on the involvement of nanotechnologies. The FDA CORES (Collaborative Opportunities for Research Excellence in Science) programme has been established to foster collaborative and interdisciplinary research on product characterisation and safety assessment. Moreover, the FDA has a specific training programme for its staff to evaluate scientific data for regulatory applications involving nanotechnologies and it conducts gap analyses with subsequent collaborative research. The outcome results in guidance documents particularly those to support the industry (US-FDA, 2020) and in the development and recognition of standards. For this purpose, the FDA collaborates with other US government departments and agencies via the National Nanotechnology Initiative (NNI) (US-NNI, 2020) and seeks a dialogue with the industry in the early product development phase. The NNI is a collaborative effort of more than 20 US departments and agencies, operating under the National Science and Technology Council of the White House Office of Science and Technology Policy. It focuses on six core topics: nanomaterials measurement infrastructure, human exposure assessment, human health, environment, risk assessment and risk management, informatics and modelling. This includes guidance documents covering topics such as workers' safety and the organisation of webinars related to characterisation and quantification of nanomaterials (Friedersdorf, 2019). The National Nanotechnology Coordination Office (NNCO) and the European Commission facilitate through the EU-US Nanotechnology Communities of Research (CoRs), a science led effort, which is open for researchers worldwide. An important project in this respect is the collaboration of the EU Nanomedicine Characterisation Laboratory (EU-NCL) – a decentralised institution composed of multiple European key expert laboratories – with its US equivalent, the US National Cancer Institute's Nanotechnology characterisation laboratory (NCI-NCL). This is important, as the accelerated development of nanotechnology-based innovative therapeutic and diagnostic products is needed to benefit patients across the globe (Borgos, 2019).

The US-FDA supports capacity building through bilateral cooperation with e.g. organisations from Canada and India and collaborates globally, especially on the characterisation of nanomaterials, amongst others through participation in technical groups of the international standardisation organisations. Considerable progress has been achieved, however, it should be stressed that the number of approved products based on nanotechnologies is still relatively low. Although there is much scientific reporting on positive findings related to the application of nanotechnology-based products in cancer research, there is the need for increased efforts to bring such products to the market. Currently, the FDA is receiving increasing numbers of submission of nanomaterials containing products, many being now in clinical trials and others having been already approved for future drugs and medical devices (Tyner, 2019).

Concerning activities on nanoplastics, there is an informal US Nanoplastics Interest Group of over 20 US agencies. This group is interested in leveraging advances learnt from nanotechnology, building on relationships and mechanisms for collaboration, and focussing on addressing current concerns and preventing contamination of the environment and food chain.

The goal of the FDA Europe Office (located in Brussels, Belgium and Amsterdam, The Netherlands) is to foster collaboration and share knowledge and information with the FDA's counterpart regulatory authorities in Europe. This is not only limited to the EU, as it includes Russia and central Asia. This Office supports primarily overall regulatory cooperation between the US and the EU, focussing on policy coherence, regulatory dialogue, policy analysis, and strategic engagement (US-FDA Europe US Food Drug Administration FDA Europe Office, 2020). Emerging technologies have been amongst the recent work areas of the Office as highlighted in the EU-US Mutual Recognition Agreement (MRA). Other topics were on the agreement on pharmaceutical good manufacturing practice (GMP) inspections, collaboration with the European Medicines Agency (EMA), holding partnership with EFSA, on medical devices and export certifications. The MRA was the result of the culmination of five years of close collaboration between the FDA, EC, EMA and EU Member States, meaning that the US and EU can now rely on each other's pharmaceutical GMP inspections avoiding duplication and allowing reallocation of resources towards priority public health risks around the globe (Nalubola, 2019).

### 3.3 Canada

Several Canadian departments and agencies carry out projects for the safe use of nanotechnology but also on risk assessment of nanoplastics, including Health Canada, Environment and Climate Change Canada, Agriculture and Agri-Food Canada and the Canadian Food Inspection Agency. These organisations adopt a regulatory focus on risk mitigation, the establishment of an inventory of products on the market containing nanomaterials, and the assessment of biological effects of specific nanomaterials. This includes the understanding on how nanomaterial products are used or consumed, considering lifecycle and exposure. The Canadian Government's Chemical Management Plan (CMP) requires actions on potentially harmful new and existing substances, including nanomaterials.

The Canadian nanomaterial regulatory approach follows the OECD (Organisation for Economic Co-operation and Development) Council recommendation on the safety testing and assessment of manufactured nanomaterials (OECD, 2020; OECD, 2020b) for regulation under the Canadian Environmental Act. The Canadian Food and Drugs Act, Consumer Product Safety and Hazardous Products Act, Pest Control Products Act, Feeds Act, Fertilisers Act, and the Health of Animals Act can also be applied in the frame of nanomaterials. Tools within the CMP include Health Canada's working definition on nanomaterials (Health Canada, 2020). As there is a lot of similarity regarding the US and Canadian definition of nanomaterials and a close regulatory collaboration via the Canada-US Regulatory Cooperation Council, the relevant departments work closely together to develop a common approach to prioritise actions and to conduct common nanomaterial risk assessment. This includes a common classification scheme for nanomaterials based on their chemical composition and unique properties. This scheme will allow for the identification of nanomaterials that behave differently from non-nanoscale products. The evaluation of nanomedicines is carried out in the Canadian National Centre of Excellence in Nanomedicines by targeting drug delivery, gene therapy and diagnostics. Canada has long been at the forefront in the development of nanomedicines for chemotherapy and gene therapy. It has been revealed that non-viral nanoparticle systems are beneficial in delivering the genetic information, facilitate the manufacturing process and have lower costs. Although research results are promising, there are still many regulatory challenges, particularly where drugs are individualised to patients or small groups of patients (Cullis, 2019). Through the OECD activities, Canadian agencies work also in close collaboration with regulatory EU agencies.

### 3.4 Asia

Regulatory science in Japan aims to achieve the best outcome for human health and society, set by the appropriate healthcare policy with the objective to transfer quickly research outcome into practical applications. The responsibility for regulation and regulatory science in this area lies with the Japanese Ministry of Health, Labour and Welfare (MHLW), the Pharmaceutical and Medical Devices Agency (PMDA), the National Institute of Health Sciences (NIHS), and the Agency for Medical Research and Development (AMED). Within the PMDA, a Regulatory Science Centre was established in 2018 to promote – amongst other – innovative approaches to advanced therapies and technologies including nanotechnologies with a major focus on horizon scanning to enable regulators to keep pace with new developments. There is a robust effort in using clinical data and data from electronic healthcare records to identify adverse events and to support product development and evaluation in large populations. Research is at the core of all activities in this integrated system. Regulatory science research on cutting-edge pharmaceutical products has been intensified by the Act on Promotion of Healthcare Industries and Advancement of Healthcare Technologies.

In 2019, the Indian government has released guidelines for the evaluation of nanopharmaceuticals which cover the scientific rationale for developing such new drugs and the comparison with existing drugs to achieve - via *in vitro* and *in vivo* studies - improved safety, efficacy, reduction in toxicity profile, reduction in required dose or frequency of administration, improvements for patients, cost benefit, and/or other benefits (Dinda, 2019).

Nanomedicines is an area of research of the National University of Singapore, providing support to drug developers and regulatory bodies in clinical and laboratory testing by identifying the critical quality attributes of the nanomedicines from clinical data sets, and determining which *in vitro* and *in vivo* methods can provide relevant markers for clinical performance. Important challenges include the accurate prediction for the elimination of long-circulating liposomes, the problem that even increasingly complex *in vitro* models do not yet improve predictions of the clinical performance, and the limited data available in the literature with regard to characterisation of nano-carriers (Wacker, 2019).

### 3.5 Chile

Chilean researchers have identified and investigated 382 research papers or patents related to plastics and microplastics legislation worldwide using keywords such as regulation, legislation, plastic, microplastic, pollution, management and policy (Velasquez Cumplido, 2019). This study revealed that solutions using circular economy models are being sought for the plastics issue, not least the recycling of materials and banning of plastic bags. Chile has been the first South American country to initiate a ban in 2018. Similar to a number of citizen science projects run in the EU, such as the European Plastics Pirate Project (European Union, 2020), many school children on the mainland Chile and Easter Island are involved in the so-called ‘National Sampling of Small Plastic Debris’. Chile is planning to generate a baseline of micro- and nanoplastics in the marine environment and their levels in fish close to fish farming centres in the south part of Chile.

### 3.6 International

The International Pharmaceutical Regulators Programme (IPRP) – under the umbrella of the International Council for Harmonisation of Technical Requirements for Pharmaceuticals for Human use (ICH) – identifies and addresses emerging issues of shared interest in pharmaceutical regulation (International Pharmaceutical Regulators Programme, 2020). Its scope covers nanomedicines, nanomaterials in drug products, borderline and combination products and methodologies used for the development and valuation. The membership of the specific IPRP nanomedicines working group includes government and agency representatives from countries in the Americas, Asia, Europe and Oceania. Its objectives are non-confidential information sharing, regulatory harmonisation, collaboration between international regulators on the organisation of training, and outreach to communities of innovators in nanomedicine and other stakeholders (Johnston, 2019).

It should be finally mentioned here that what actually needs to be reported on nanomaterials to regulators, and in which metrics (i.e. based on particle numbers or weight), depends on the regulation which is applicable in specific sectors and jurisdictions. For example, particle size distribution based on particle numbers is needed for identification of nanoforms according to the REACH legislation in the EU, whereas the weight fraction of nanoscale particles determines whether reporting of a material to the US EPA is required under the US Toxic Substances Control Act (US EPA, 2017). Sometimes both metrics are required, for example for notification of a nanomaterial in the European Commission's Cosmetics Products Notification Portal (CPNP).

## 4 Nanotechnology in the agriculture/food/feed sector

Nanomaterials can be intentionally used as/in food formulations or may be unintentionally present especially when it comes to their powdered form. A prominent example is titanium dioxide. In the EU, as food additive, it must be labelled according to provision of Regulation (EU) No. 1169/2011 (European Union, 2011). If present also in the form of engineered nanomaterials, according to article 18(3) of this Regulation, this shall be clearly indicated in the list of ingredients. In this case the name of the ingredient shall be followed by the word ‘nano’ in brackets (Geiss et al., 2020).

The benefits of nanotechnology for the production of food ingredients using encapsulation include improved bioavailability and/or absorption; masking undesirable tastes or flavours; achieving controlled release of the encapsulated ingredients (e.g. of vitamins or minerals); protecting the encapsulated substances from the external environment; and achieving controlled interaction between the encapsulated ingredient and the food matrix. There are, however, many variables in such nano-sized delivery systems. Some structures function well in water but are not necessarily transferable to other food matrices. The delivery systems need to be food grade, safe and compatible with the food matrix. The release of the encapsulated ingredient is dependent on many variables, including the shape and dimensions of the delivery system; its chemical composition; the type of ingredient and its diffusivity and solubility in the encapsulant; the ingredient ratio in the delivery system and the external medium; and the encapsulation load and the loading efficiency. The release mechanisms of nano-sized delivery systems results in various processes of diffusion into the human body amongst others the degradation by digestive enzymes. While there are several products on sale already, there is a strong motivation to develop optimal formulation. The aim is to use less ingredients and having an improved bioavailability. It is of great importance to have information on potential risks at hand before nano-sized materials find widespread application in the food sector. It is yet not clear whether nano-sized materials are digested differently than their respective course or bulk materials. In case the absorption and bioavailability of nano-sized/encapsulated nutrients can be improved, dietary modifications may be needed to arrive to accepted daily intakes for the materials in their nano-form. There is an urgent need for *in vitro* and *in vivo* experiments to study the efficacy of the delivery systems as well as potential adverse effects (Greiner, 2019).

The key driver for applying nanotechnology in the plant protection area is to address the weaknesses of existing products. Currently authorised and applied pesticides can be improved through three main avenues: more efficient application of stable suspensions giving homogenous coverage; reduced losses and transportation to non-target areas; and improved interactions with the host or with the target (e.g. improved uptake). To-date there is already a wide variety of nano-enabled pesticides on the market, the first generation including metals and metal oxides and the second generation being nano-carrier loaded with an active substance. The latter can be, for example, an existing pesticide (and the nano-pesticide is therefore a reformulation), a novel nano-pesticide or nature-inspired nano-carriers. The durability of a nano-pesticide is how long it maintains the properties that are specific to its nano-size. Nano-pesticides can have different size distribution or release rate. The measurement approaches are currently limited, and more work is needed to characterise nano-pesticides over time after they were applied in the field (Kah, 2019). Besides nano-pesticides, a number of specific projects investigate the efficiency of nutrient use in agriculture by using nanoscale elements not only to suppress plant disease but also to increase crop yields (Chen, 2019).

Another application of nanomaterials in the agriculture/food/feed chain is to reduce waste. Besides many other causes, food waste can be due to bacterial, chemical or fungal spoilage. The early and rapid detection of e.g. pathogens is not only of utmost importance for food safety and public health, but also for the food processing industry. Spoiling sensing devices use increasingly nanomaterials such as in metal oxide semiconductor gas sensing technologies, finding application in the meat industry. The sensitivity of sensors is enhanced by using nanomaterials offering a higher surface area per volume of sensing material. Nano-enabled devices are also being developed for the rapid, sensitive and selective detection of e.g. salmonella and hepatitis C pathogens. Such nano-sized biosensors can reduce the time for detection from days to a few hours, thereby also reducing the costs (Bosnick, 2019). Research is ongoing on printable nano-based sensors for the detection of pathogen residues in the agricultural fields and on the different behaviour of the human body in comparison to control animals (Chen, 2019).

Nanocomposites can also be used for (renewable and degradable) food packaging. While blending, for example, cellulose nanocrystal nanorods with renewable-derived packaging adds only passive reinforcement, smart functionality can be obtained by synthesising designer hybrids with active materials. This can lead to smart, renewable packaging that can sense meat spoilage, by e.g. using zinc oxide nanoparticles as the means to optically sense biogenic amines.

As already mentioned before, according to EU legislation, nanomaterials are chemical substances and are regulated by REACH (EC Regulation 1007/2006) with EC Regulation 2018/1881 modifying the REACH annexes to include nano-specific clarifications and requirements. The European Commission recommendation on the definition of nanomaterials (European Commission, 2011) – as already discussed in Section 2 – provides a definition of the term ‘nanomaterial’ across all EU legislation. Regarding nanotechnology in the food chain, which is governed by food legislation, guidance from EFSA was made available in 2011 and updated in 2018 (EFSA, 2020b). EFSA prescribes and evaluates the adequacy of risk assessments for the relevant application dossiers. Those mainly concern the two areas of food contact materials and food additives, but also application for feed additives and novel foods have been submitted to EFSA. EFSA currently works on a more detailed technical guidance for applicants concerning the physico-chemical characterisation, which will, inter-alia, enable EFSA to provide criteria for understanding of the presence of a nano-fraction in a product, as there is a scarcity of data specific and relevant for nanoparticles. It should be stressed that all new products are subject to the current legislative framework; however, there are products on the market, which were released when no nano-specific guidance was available. When those products come up for renewal, applicants will have to demonstrate the safety, taking into account the current EFSA guidance. The guidance covers areas such as novel foods, food contact materials, food and feed additives, and pesticides, and is intended in particular for risk assessors, risk managers and applicants. Hazard identification and characterisation must take into account that a nanomaterial may lose its nanoform in food preparations or when arriving the digestive tract of the human body. At this point, the nano-specific guidance would no longer be relevant (Schoonjans, 2019). To cope with these many challenges, EFSA has a crosscutting working group providing expert advice to EFSA staff and panel experts (EFSA, 2020). It works with stakeholders in the EU Member States through the EFSA Nano Network.

As already discussed, the physico-chemical characterisation of nanomaterials is a key process in risk assessment together with the quantification of nanomaterials in food and feed for exposure assessment. Besides a considerable number of parameters including particle size, particle shape, elemental composition, crystal structure, surface charge, surface area and agglomeration or aggregation state, there are also other properties to be considered such as physical and chemical stability of nanomaterials and coatings; solubility in relevant media; dispersibility of poorly soluble nanomaterials; and chemical reactivity/catalytic activity. The sample preparation in the analytical process is challenging (Correla et al., 2019) and it is important to note that a single analytical method may not be sufficient. So far, there are no generic methods for the characterisation of nanomaterials, but there is a large variety of individual characterisation methods available, with different capabilities and fields of application. A thorough knowledge of the measurement techniques' capabilities and detailed information of the materials’ physicochemical properties allow selecting the method(s), which is/are best suited to measure the attributes of a material with specific physico-chemical properties (Mech et al., 2020a, 2020b; Rauscher et al., 2019b).

Advanced analytical methods need to be developed that are specific to certain nanoparticle/matrix combinations and sometimes even for specific nanoparticle characteristics (e.g. surface properties) (Mech et al., 2020b; Rasmussen et al., 2019a; Loeschner, 2019). The need for harmonisation/standardisation of analytical approaches will be further discussed in Section 6.

As nanomaterials of the same core chemistry can exist in many different forms (i.e. shape, surfaces, sizes), it would be desirable to apply grouping of substances together with the use of data from structurally related substances (read-across) for risk assessment (Stone et al., 2020; Giusti et al., 2019). However, to-date, grouping and read-across are rarely applied in the food sector. Specific hurdles need to be overcome in putting nanomaterial grouping into practice include the very low number of case studies of this approach. There is only limited mechanistic information available and reliable *in vivo* data are scarce. There is still a lack of systematic, comparative data sets with uncertainties resulting from insufficient data quality and missing benchmark materials. Moreover, risk assessors face difficulties in correlating the physico-chemical properties with toxicity due to the uncertainties about the relevant parameters and variations due to life cycle modifications, e.g. where the material changes during use that may result in changes in its toxicological behaviour (Herzberg, 2019). Such challenges have been identified when assessing the potential risks deriving from aluminium nanoparticles. Aluminium is a widely found packaging material in direct contact with food and can occur in food additives, flavourings and processing aids. The current data on aluminium-containing nanomaterials is limited and insufficient for an appropriate safety assessment. It is important to understand how the different forms of aluminium behave in the human body. It has been found that *in vitro* results did not mimic what was observed *in vivo*. The nature of the link between genotoxicity and accumulation has shown to vary according to which organs and tissues were examined (Fessard, 2019). To address these challenges, various national and international initiatives (projects) provide/provided information that can be accessed through specific databases such as eNanoMapper (Jeliazkova et al., 2015). It needs to be stressed that agreement on data quality requirements is key, requiring more research for the development of comparative datasets based on standardised and validated methods (Comandella et al., 2020) and including also next-generation nanomaterials (Herzberg, 2019; Fessard, 2019). These challenges associated with grouping and read-across are tackled by the H2020 project GRACIOUS which has already developed a highly innovative framework for grouping and read-across of nanomaterials thereby potentially reducing the need to assess exposure to and toxicity on a case by case basis (Stone et al., 2020).

## 5 Examples and challenges on safety assessments of nanomaterials

Although products derived from nanotechnologies and nanomaterials have already reached the global market, there is still the need to understand the fate and effects of such materials in the environment, in biomedical applications and other consumer products. Many efforts in the US, the EU and other organisations worldwide have addressed and are still working on the safety assessment of nanomaterials. Although there are many ongoing international efforts in this respect, e.g. the work carried out by specific OECD working parties (OECD, 2020), there is still much to achieve and to understand. This Section therefore summarises the actions of government agencies and research bodies across the globe aiming at the harmonisation of the assessment of nanomaterials via test guidelines standards and risk assessment frameworks.

Concerning the impact of nanomaterials and nanotechnology derived products on the marine environment, the US Environmental Protection Agency (EPA) has a specific research programme to understand, amongst others, the levels of toxicity and bioaccumulation. It has been found that salinity is a key driver affecting nanomaterial stability and their bioavailability (Burgess, 2019). Whereas large agglomerated nanoparticles tend to settle out in the sediment, resulting in fewer concerns for the marine ecosystem, some toxic ions have been seen to leach out from some types of nanomaterials into water, resulting in adverse effects. It is however important to have the right analytical methods at hand to extract and identify nanomaterials from the marine ecosystem. This is challenging, as manufactured nanomaterials are often mixed with natural materials, making it difficult to assess them in the marine environment compared to the quantity of naturally occurring nanomaterials. The toolbox for measuring adverse effects in the marine environment is also limited and methods generally focus on acute toxicity, while sub-lethal toxicological methods are also needed (Burgess 2019).

ECHA, in close collaboration with EFSA – dealing with safety assessment of nanomaterials in the food and feed chain - and other bodies of the European Commission, including the JRC, is finalising guidance documents, tools and methods in this respect. The guidance relies on available (and ideally standardised) methods, a focus area of the JRC in collaboration with international bodies including the OECD, the European Committee for Standardisation (CEN) and the International Organisation for Standardisation (ISO). ECHA assesses the incoming substance registration dossiers (to date about 40 containing information on nanoforms) case by case to understand the robustness of the data included and its completeness. As already mentioned in chapter 3, ECHA also hosts the European Union Observatory for Nanomaterials that aims to provide objective and reliable information to the public on innovation and safety aspects of nanomaterials on the EU market (ECHA, 2020).

As mentioned above, the EU Cosmetics Regulation No. 1223/2009 (European Union, 2009) covers also the safe use of nanomaterials as ingredients in cosmetic products. A prominent example are sunscreens containing titanium dioxide in its nanoform with the aim to provide a high level of protection for human health. There are specific regulatory provisions for nanomaterials such as notification to the authorities six months before market release. The EU Scientific Committee on Consumer Safety (SCCS) addresses among other safety concerns that may relate to the crossing of membrane barriers in the human body, accumulation and bio-persistence. However, to-date there are no complete safety dossiers addressing nanomaterials in cosmetics, but the SCCS has issued opinions on nanomaterials as cosmetics ingredients for various applications (European Commission, 2020; De Jong, 2019) and the last revision of the SCCS Guidance on the safety assessment of nanomaterials in cosmetics was published in 2019 (SCCS, 2019). However, there are still some uncertainties and knowledge gaps with regard to physicochemical properties, environmental behaviour and toxicological effects, especially as testing described in the dossiers is often done early in the product development process, and the material in the final product may behave differently. Therefore, the main challenges for dossier evaluation of nanomaterials used as cosmetic ingredients include the following needs: high quality characterisation of the materials; to understand the uncertainty whether the materials described and presented in the dossier is the same as the material that was actually tested for safety; to take into account the possibility for internal exposure due to translocation of the nanomaterials; to include non-animal testing methods (as required for the production and marketing of cosmetics in Europe (European Union, 2009); and to understand what needs to be a robust weight of evidence approach to safety assessment.

A recent detailed study on the characterisation of titanium dioxide used as food additive in the confectionary products has not only revealed the importance of the most appropriate analytical method for detection and quantification, but moreover also the need for appropriate sample preparations methods (Geiss et al., 2020). It should be stressed that this is relevant for the characterisation and safety assessment of all nanomaterials.

The safety assessments and evaluation of nanomedicines by EMA include liposomes, iron nanoparticles, nanocrystals, albumin nanoparticles, and lipid nanoparticles, with many more types of systems under research (Genchi et al., 2017; Perez de la Ossa and Bremer-Hoffmann, 2019). As the term ‘nanomedicine’ covers a wide variety of materials and structures, with unique and distinct features, there is no ‘one size fits all’ for evaluation of the products. EMA supports drug developers to launch safe and beneficial products through guidance documents (EMA, 2020). It is important to pay early attention on the critical quality attributes concerning the drug's activity and safety, the identification of appropriate analytical characterisation methods, and ensuring that scale-up to a reproducible large-scale manufacturing can be achieved.

In the US, the safety assessment of medical devices containing or deriving from nanotechnology is carried out by the US-FDA's Centre for Devices and Radiological Health (CDRH), housing a Nanotechnology Regulatory Science Research Programme that is based on three pillars: physico-chemical characterisation methods, *in vitro* and *in vivo* models, and (toxicological) risk assessment. The types of devices that incorporate nanotechnology include antimicrobial, dental, orthopaedic, neurological and combination devices, and *in vitro* diagnostic tools. They use various nanomaterials, including silver, zirconia, titanium and titanium dioxide, iron oxides, polymers, gold, graphene etc. Safety assessment of such medical devices should encompass the determination of the rate and magnitude of the nanomaterials into the body for which fit-for-the-purpose *in vitro* tests would be desirable. Moreover, advanced toxicological risk assessment approaches should support the understanding of the release and patient exposure results in adverse health impacts. It is important to know whether nanoparticles affect the accuracy and/or reliability of standard biocompatibility or toxicity test assays, such as for cytotoxicity and genotoxicity. Because of the vast number of sizes, shapes, and chemistry of nanomaterials, there is the need for the development of *in vitro* models (2D, 3D, organ on a chip, organoids) and *in silico* models in order to predict human responses and improve *in vitro* to *in vivo* extrapolations (Goering, 2019).

One focus of the Japanese NIHS is on the risk assessment of silver nanoparticles as they are used in many kinds of consumer products, including food contact materials, textile fabrics and cosmetics due to their antimicrobial properties (Ogawa, 2019). Animal studies on various silver nanoparticles (10 nm, 60 nm, 100 nm) have shown that the degree of toxicity is not defined by the total surface area but moreover by the size of particles, with silver nanoparticles of 10 nm size being the most toxic as ionic silver could be observed in the liver of the treated animals (Boudreau et al., 2016). It is of utmost importance to carry out risk assessment using doses that relate to the real environment (most likely much lower than used in many research studies). Moreover, there is a need for more data on excretion and accumulation of nanomaterials when assessing their possible risks.

Safety assessment of nanomedicines is likewise important. Examples of nanoparticles used by pharmaceutical companies include polymeric nanoparticles encapsulating drugs that are released through diffusion and polymer-conjugated drugs released via linker chemistry (dendrimers). In order to understand their behaviour in physiological conditions, it is necessary to explore the aggregations and solution behaviour of nanoparticles. This is critical for dendrimer drugs, where the active pharmaceutical ingredient and the dendrimer (nanoparticle) are covalently bound, and the release profile of the dug depends on the composition of the dendrimer, and on time. The drug release profile from encapsulated drugs, e.g. accurins, is more complex. The drug molecules in the centre of the nanoparticles are seen to disappear the slowest, as they have the furthest distance to travel to escape the nanoparticle. Moreover, the size of the nanoparticles seem to affect the drug release as well. *In silico* simulations are used for optimisation of the release behaviour. The controlled release from a particle can change from the initial stages, where there should be uniform concentration of the active component, to late stages, when some molecule have been released and the concentration and distribution of drugs are different (Akhtar, 2019). The challenges in biopharmaceutics are huge, as a change of one parameter can affect many different characteristics and can fundamentally change the medical formulation under investigation. Therefore, robust nanomaterials characterisation methods are of utmost importance to enable bioequivalence to be demonstrated together with the understanding of the impact of process parameters on product attributes. This should be underpinned by *in vitro* and *in vivo* correlations with biopharmaceutical bridging strategies to secure flexibility for a complete product Akhtar, 2019).

Complex drugs derived from bio-and nanotechnologies have a huge potential in the treatment of severe diseases such as cancer and anaemia. However, as the manufacturing process defines their characteristics, it is challenging to ensure homogeneity affecting the development of follow-on drugs and their evaluation for marketing authorisation (Schellekens et al., 2011). The main regulatory challenges are the lack of standardised nomenclature and test methods for safety assessment (De Vlieger, 2019).

Using a polyamidoamine dendrimer model it is possible to investigate the blood and endothelial toxicity of engineered nanoparticles. Researchers from the FDA focused on the effects of nanomaterials on blood platelets and the cells lining arteries and veins (vascular endothelial cells). It is important to understand what assays need to be used to predict any potential adverse effects (Simak, 2019). The challenges in the evaluation of the effects of nanomaterials on blood coagulation and the main concerns for these assays are: appropriate sample preparation; characterisation of the nanomaterial under test conditions (size distribution, particle aggregation, surface charge, etc.); nanomaterial dosimetry (which should be based on the surface area); appropriate selection of assays for different purposes (e.g. screening and mechanism investigation); and the need for standardisation and inter-laboratory studies. The latter is challenging as it is of utmost importance to obtain healthy cells for the studies and dealing with individual variability of donors’ blood (plasma, platelet) samples, resulting in the need for a large sample size to reach satisfactory reproducibility (Simak, 2019).

The potential risks of bionanotechnology derived products (e.g. DNA- and RNA-origami) are not known yet, given the relatively unknown kinetic (pharmacokinetic and pharmacodynamics) behaviour of bionano structures or on the uptake by the human body. In vitro it is seen to be taken up by the cells, although its degradation path and products remain unclear. Hazards include that such foreign DNA, RNA and proteins may evoke an immune response, although that could be the desired trait for the future design of adjuvants for vaccines. Therefore, many knowledge gaps need to be resolved for these new forms of materials (Oomen et al., 2019; Oomen, 2019).

Although there are many national and global actions taken to-date on safety assessments of nanomaterials, it can be concluded that risk assessment approaches are still incomplete due to the vast number of nanoparticle sizes, shapes, chemistries and surface functionalities. Although the results of recent studies can be used to develop tolerable intake values for the clinically relevant routes of exposure (oral, inhalation, dermal etc.), there are still gaps regarding the availability of robust assessments for exposure and dose-response. The determination of the margin of safety remains difficult with the current trend of reducing the number of animal studies. Alternative to animal testing methods need to be further developed and incorporated into safety testing approaches. Regulatory science should not neglect nanomaterials dosimetry (metrics of mass, surface area and number) and needs to ensure that doses used in risk assessment are of clinical relevance, but accurate measurement methods of these parameters in products is still elusive. Moreover, there is a need for intensified academic and regulatory science collaboration worldwide. Harmonisation of risk assessment methodologies and transparency of the outcome of regulatory science are important tools. The already worldwide collaboration on proficiency testing and method validation should be increased, however, the topic is challenging due to the lack of test (reference) materials that can serve as positive or negative control samples (see more details in Section below).

## 6 Challenges concerning nanoplastics

As discussed in the previous Section, there is a huge effort by scientists and regulatory bodies to understand the benefits and potential risks of nanomaterials/nanotechnologies to be applied in various areas. For manufactured nanomaterials (including polymer-based materials) there is a lot of progress in the risk benefit analysis, and, if needed, some classical risk management measures can be applied. Although there are still many challenges to address and overcome, those are much more prominent when dealing with micro- and nanoplastics. It can be assumed that by far, the largest share of these plastic particles in the environment derives from the degradation of plastic products rather than from intentionally added microplastics to products. There is to-date only limited information on synthesised nanoplastics (Nguyen and Tufenkji, 2020), but there is no information available regarding the stability of secondary nanoplastics. As tiny scale particles in the environment especially due to their aged character may easily permeate with oxygen or water, a fast degradation respectively decomposition of microplastics and nanoplastics can be expected. A study on the reactivity and potential impact of nanoplastics on atmospheric and surface waters (using polystyrene nanoparticles as proxy) showed that smaller nanoplastic particles have a higher reactivity with hydroxyl radicals and that the degradation of nanoplastics releases organic compounds in the aqueous phase (Bianco et al., 2020). Therefore, there is an urgent need for more appropriate waste management to minimise pollution. Moreover, considering the current knowledge, ECHA concluded that the release of intentionally added microplastics can pose a risk that is not adequately controlled. At the same time, it is acknowledged that the current data provides an incomplete picture of the situation. A significant issue is the lack of a consistent and widely accepted/harmonised definition of the terms ‘microplastics’ and ‘nanoplastics’. The proposed restriction of intentionally added microplastics and nanoplastics includes different regulatory measures: prohibition on placing on the market; derogated uses (for natural/biodegradable polymers and uses without foreseeable microplastics release; labelling obligations (for uses where microplastic release can be minimised with instructions for use); and mandatory reporting requirements (of identity, description of use, tonnage, releases). Outside the scope of the ECHA restriction would be materials that occur in nature and have not been chemically modified, or materials that are (bio)degradable (Bintein, 2019; ECHA, 2019).

Whereas many efforts are now undertaken to further develop methods for the determination of microplastics – including ongoing inter-laboratory studies – and to better understand the exposure to micro-sized particles, there is currently no adequate analytical methodology available to detect and characterise nanoplastics. Although the presence of nanoplastics in nature is generally considered highly plausible, there is still little or no information on their presence in freshwater, soil or the atmosphere, in biota, drinking water or food. Studies on microplastics have shown, for example, adverse effects in duckweed, algal photosynthesis, marine biota, molluscs and copepods (SAPEA, 2019). Nanoplastics are assumed to pass biological barriers such as the gut epithelium. However, the studies performed in this area have used much higher concentrations of plastics than those currently reported in the environment – though data on the latter are still uncertain, again due to the lack of reliable analytical methods. Moreover, the few reported studies mainly used spherical particles that do not represent ‘real-world’ particles, or were conducted using relatively short exposure times. To-date it is unclear whether nano- and microplastics cause toxicity in marine organisms (GESAMP, 2016; UN-Food and Agricultural Organisation, 2020) and whether such plastic debris may present an attributable risk to human health when exposed to environmental contamination or through the food-chain (Hantoro et al., 2019; United Nations, 2016).

Besides their main constituent, i.e. the polymer(s), plastics may contain also small organic molecules (additives, residual monomers, polymerisation aids) that can leach out into food and the environment once nano- and microplastics are released. At the same time, the debris plastic material can take up other hydrophobic organic chemicals from the environment. Laboratory studies indicate that those substances can be transferred from plastics to tissues of various organisms. However, the evidence today is weak to support the occurrence of ecologically significant adverse effects on aquatic life. As already mentioned above, there is a gap between ‘real’ environmental exposure (in terms of concentrations, sizes, shapes, polymer types, weathering states) and exposure in laboratory-based studies, hampering sound risk assessments and not yet reliable in practice (Hartmann, 2019).

Nanoplastics have been found to be highly mobile in soil and the organic substances therein seem to have a strong influence on the fate of the plastic debris. Plastic additives may increase transportation efficiency while increasing size and asymmetrical shape may be linked to increasing deposition. Caution has to be taken against extrapolation from one nanomaterial (a specific nanoplastic) being a potential hazard to all nanomaterials (all nanoplastics) being dangerous (Gigault, 2019).

There is an urgent need for reliable data on concentrations and properties of nano- and microplastics in the environment and the food chain. There have been reports on the occurrence of plastics in a variety of food products including bivalves, salt and mineral water (Van Cauwenberghe and Janssen, 2014; Karami et al., 2017; Zhang et al., 2020; Schymanski et al., 2018). Small plastic debris may derive from plastic packaging, but also from manufacturing processes. Although microplastics occur in food (Toussaint et al., 2019), there is – as already discussed in Section 2 - no harmonised definition of microplastics (in terms of size and morphology) and no appropriate and validated method of quantification. Since risk depends on the potential hazard, the exposure and the uptake, there is insufficient data to make a clear risk assessment (Lynch, 2019; Henry, 2019). It is necessary to employ multiple methods for detection and characterisation, and methods available to date for microplastics may not be appropriate to detect and quantify nanoplastics (Lampen, 2019).

Current evidence about ecotoxicological effects of micro- and nanoplastics is limited. However, if environmental emission rates remain unchanged, ecological risks of these particles may be widespread within a century. More (quantitative and qualitative) information is needed on environmental and human exposure to nanoplastics, enabled by fit-for-the-purpose and ideally harmonised measurement methods. Comparing micro-to nanoplastics, it has been found that nanoplastics are physically less likely to settle than microplastics and they may be transported over longer distances than larger plastic particles. Nanoplastics are also thought more likely to enter the food chain (Al-Abed, 2019).

Considering whether existing nanoparticle analytical methods could be used for nano- and microplastics, it is important to note that many engineered nanomaterials are inorganic in nature and more easily identified and quantified. In contrast, micro- and nanoplastics being mostly composed of the same elements as biologic matrices (carbon, oxygen, hydrogen, nitrogen) are more difficult to be unambiguously identified and determined. As for inorganic nanoparticles, a reliable experimental identification and quantification of a micro- and nanoplastics particle distribution requires analysing a sufficiently large number of particles. Integral or ensemble methods in principle measure large numbers of particles simultaneously and their composite signal is used to extract information on the particle size distribution. However, this advantage is contrasted by loss of information on individual particles and often a very strong bias towards detection of larger particles. Counting or imaging techniques investigate particles individually but require much more time to analyse a sufficient number of particles (Rauscher et al., 2019b). There are some established analytical methods that can be applied for identifying microplastics although there is a need for further method development, and, above all, harmonisation and standardisation. For the smaller-sized nanoplastics, these methods are not effective and need urgent method development (Gilliland, 2019).

Research on microplastics and especially on nanoplastics has started only recently and thus, there are many unresolved issues of definitions, sampling, characterisation and the assessment of hazard and exposure that, in combination, make it very difficult, to evaluate and regulate the potential risk of nanoplastics. While some information on microplastics is available, research data on nanoplastics is virtually non-existent. Coordinated efforts are needed across all these areas in order to produce reference materials that mimic the real-world mixtures, robust standardised methods, guidance and legislation. Such efforts can and should build upon the existing knowledge and lessons learnt from the area of nanomaterials, and with existing national and international mechanisms, including governmental and intergovernmental bodies. Focus should be on the complex mixtures assessment, from characterisation, hazard, exposure and risk assessment, and should utilise lessons learned from decades of nanomaterials research. A platform for information exchange on nano- and microplastics is needed, along with the global coordination of activities on methods, documentary and material standards towards hazards, exposure and risk assessment to discuss and ascertain effects on the environment and human health.

More work needs to be done to determine the extent to which nanoplastics are formed in the environment and to understand where those particles would accumulate. It is important to clarify the relationship between the morphology, behaviour and impact of the particles, including the understanding whether some shapes of nanoplastics (e.g. fibres) are particularly problematic to environmental and human health. The understanding where and how nanoplastics are formed and released, e.g. via tyre abrasion or washing of synthetic textiles, is of major importance for the conception of appropriate regulatory mitigation measures. This implies that determination of plastic mass or number of particles is not sufficient, but that information about the chemical identity and form is needed.

## 7 Documentary and material standards

Standards are the consolidation of well-established knowledge meaning that standardisation is generally not a primary way of sharing new research information. The developer usually funds the development of standards. Documentary standards deal amongst others with classification and terminology, provide guidance and good practice and describe test methods and analytical approaches, which are agreed upon the principle of consensus. Material standards, on the other hand, are physical samples used as a reference with a stated property and, if appropriate, a measurement uncertainty.

Due to the fact that to-date there is only limited knowledge about the analytical performance of methods used for the detection, quantification and hazard identification of micro- and nanoplastics, this chapter deals mainly with the harmonisation and standardisation related to nanotechnology/manufactured/engineered nanomaterials.

There are many complementary efforts related to the development of documentary standards especially in the field of nanotechnology/nanomaterials. From GSRS 2016, it was concluded that, for regulation of nanomaterials, appropriate documentary standards are needed (GCRSR, 2016). These include standards for physicochemical characterisation of nanomaterials (identification and quantitation, quality attributes considered critical for appropriate regulation, such as particle size and surface properties, and common methods), *in vitro* assessment (e.g. interaction of nanomaterials with the immune system, drug loading and release from drug delivery systems) and other considerations such as materials that are already widely used (e.g. liposomes) and characterisation in biologically-relevant media. Standardisation organisations are very actively working on developing such documentary standards applicable for nanomaterials and nanotechnology. The ASTM International, for example, has taken action addressing some of these regulatory needs identified at the GSRS 2016, albeit with many standards being work in progress as it takes some 12–18 months to draft a standard document. The process of developing a standard is lengthy, as standardisation requires extensive inter-laboratory studies. ASTM International aims to avoid duplication of work of other standardisation bodies. Within ASTM, ASTM E56 Committee is working on nanotechnology.

The International Organization for Standardization (ISO) is a non-governmental international organization that develops consensus-based, international standards. Specifically, the ISO Technical Committee (TC) 229 is working on standardisation in the field of nanotechnologies. ISO TC 229 welcomes official liaisons and invites other standardisation bodies for collaboration as described below (Koltsov, 2019). Still, it can be difficult to avoid duplication, as ASTM work is proposed by individual members, for example, a company or regulatory agency, but some other standardisation bodies, e.g. ISO or the OECD, may already have a similar activity in work (Kaiser, 2019).

The OECD Test Guidelines (TG) are regulatory recognised methods used for safety assessment of chemicals including nanomaterials (OECD, 2020b). OECD TGs fall under the system of Mutual Acceptance of Data in the Assessment of Chemicals (MAD), which means that test results obtained following an OECD TG are legally accepted in OECD member countries and other adherents. MAD is established for industrial chemicals, pesticides, biocides, food and feed additives and cosmetics. It aims to reduce duplication of testing and use of animals, and to lower trade barriers. A clear understanding of the regulatory context supports scientific and regulatory acceptance of a TG across several countries, and it is important to know the regulatory requirements before undertaking data collection. An OECD TG can be developed and published within a two-year-period, but some may take even 10 years to finalize. To facilitate the process, it is important to have early discussions between method developers and regulators, and all stakeholders need to adopt an issue-solving attitude. There needs to be clarity about the intended use of the assay or battery of assays and their purpose, e.g. for priority setting, hazard identification, or risk assessment. The obtained data should be available in the public domain (Gonzalez, 2019). Relating standards for nanomaterials, the OECD Working Party on Manufactured Nanomaterials (WPMN) works closely together with ISO. The OECD develops test guidelines for regulatory testing that are applicable to all or specific groups of chemicals or nanomaterials, not to individual ones. OECD guidelines often refer to ISO or other standards, which are either applied as such or adapted in order to implement specific requirements (OECD, 2020b).

The development process of ISO standards begins with a pre-draft document - developed and accepted as best practice at national level - through consultation with all stakeholders, which will be transferred into an ISO draft document receiving comments from all ISO members and acceptance from ISO experts. This process is typically taking two to four years, before a final ISO international document is approved. ISO/TC 229 (Nanotechnologies) was founded in 2009 and has a membership of 33 participating and 10 observing countries. So far, it has developed more than 70 standards and related informative documents and more than 40 are under development. The main scope of ISO/TC 229 is the development of standards for the terminology and nomenclature, metrology and instrumentation, including specifications for reference materials, test methodologies, modelling and simulation and science-based health, safety, and environmental practices. As mentioned above, ISO works closely with the OECD WPMN, but also with CEN and ASTM international (Koltsov, 2019). The Nanotechnologies Technical Committee 352 of CEN - founded already in 2005 - is involving 34 European countries. So far, it has developed some 15 standards with a similar number in progress, whereas ASTM International TC E56 – also established in 2005 with some 180 individual members - has published some 20 standards with many in development.

CEN has an agreement for technical co-operation with ISO. The Vienna Agreement, signed in 1991, was drawn up with the aim of preventing duplication of effort and reducing time when preparing standards. As a result, new standards projects are jointly planned between CEN and ISO. Wherever appropriate priority is given to cooperation with ISO provided that international standards meet European legislative and market requirements and that non-European global players also implement these standards. Currently almost all standards related to nanotechnologies and published by CEN/TC 352 are adopted or slightly modified ISO standards.

In China, the Standardisation Administration of the People's Republic of China (SAC) provides guidance and standards on nanotechnology via its TC 279. So far, standards have been developed for applications of silver and gold nanoparticles and carbon-based nanomaterials. More than 40 technical protocols have been developed of which 14 are in the physico-chemical field and some 30 on *in vitro* and *in vivo* biological effects. There is close collaboration with the Chinese National Medical Products Administration (NMPA) – formerly known as the Chinese FDA. Moreover, there is a joint platform between the NMPA and the Chinese Academy of Sciences (CAS) on regulatory science for innovative medicines. More international collaboration is planned on standards, characterisation and other key issues of regulatory science. As many documents are available only in Mandarin, it would be beneficial for international cooperation to translate them into English and to submit them to ISO (Xie, 2019).

The US FDA's Office of Research and Standards (ORS) aims to make safe and effective generic drugs available to the American public, and implementing standards based on the best available science. For the FDA-approved nanotechnology drug products, a concept of product-specific guidance was developed. Approved nanomaterial-containing products are based on liposomes, inorganic nanoparticles, protein nanoparticles, polymer nanoparticles, emulsions, lipid complexes, nanotubes and nanocrystals, and micelles, all products used to combat diseases such as cancer, anaemia, macular degeneration and others. The US nanotechnology standards database is hosted by the American National Standards Institute (ANSI), the U.S. member body to ISO, and contains published and government documents. The challenges regarding nanomedicines are highlighted below (Jiang, 2019).

The Canadian national Research Council (NRC) undertakes efforts to understand the science of measurement at the nanoscale for the better understanding and adoption of new nanomaterials through advanced characterisation methods and the development of standards and materials. Amongst others, the NRC is developing quantitative metrological assessment protocols for the characterisation of nanotubes and graphene related materials. A special interest group of the NRC facilitates the development of standards for graphene made by exfoliation methods. Canada actively supports the worldwide nanoscale measurements and nanomaterial metrology efforts and contributes to the international standardization bodies as mentioned above (Zou, 2019).

An in-depth discussion on international standards in nanotechnologies including detailed tables of existing ISO standards in that field is provided by Clifford et al. (Clifford, 2020). Table 2 provides an overview of active ASTM standards (Technical Committee E56). IEC TC 113 (Nanotechnology for electrotechnical products and systems) has published a number of standards, most of them on nanomanufacturing.

The OECD Working Party on Manufactured Nanomaterials (WPMN), together with the OECD Test Guidelines Programme (TGP) has explored the need for adaptation of existing OECD TGs and Guidance Documents (GDs) as well as developing new TGs and GDs to specifically address nanomaterial issues. A detailed overview of the progress made by 2019 is provided by Rasmussen et al. (2019b). Table 3 lists the newly adopted or adapted OECD TGs and Guidance Documents (GD) specifically addressing manufactured nanomaterials. Development of more nano-specific TGs and GDs and the adaptation of existing ones to address those specific issues are ongoing.

**Table 3:** *Newly adopted OECD TGs and GDs explicitly applicable to nanomaterials*.

Document number	Title	Notes
OECD TG 318	Dispersion Stability of Nanomaterials in Simulated Environmental Media	New TG in 2017
OECD TG412	Subacute Inhalation Toxicity: 28-Day Study	Updated in 2018 to address nanospecific issues
OECD TG413	Subchronic Inhalation Toxicity: 90-day Study	Updated in 2018 to address nanospecific issues
ENV/JM/MONO(2009)28/REV1 (OECD Series on Testing and Assessment, No. 39)	Guidance Document on Acute Inhalation Toxicity Testing	Updated in 2018 to address nanospecific issues
ENV/JM/MONO(2020)8 (OECD series on testing and assessment, No. 317)	Guidance document on aquatic and sediment toxicological testing of nanomaterials	New GD in 2020
ENV/JM/MONO(2020)9 (OECD series on testing and assessment, No. 318)	Guidance document for the testing of dissolution and dispersion stability of nanomaterials and the use of the data for further environmental testing and assessment strategies	New GD in 2020

With the rapidly developing domain of nanotechnology, it is particularly important that the work on reference materials and standards will be accelerated through better collaboration and communication. Although there are some international efforts taken by organisations such ISO, OECD, ASTM International, US National Institute for Standards and Technology (NIST), and the European Commission through efforts taken by the JRC related to method validation and reference materials, more coordination of efforts would be desirable to avoid duplication of work.

Concerning the characterisation methods for standards development, it needs to be stressed that to-date most methods are highly product-specific with multiple different methods developed by different laboratories. Moreover, many of the advanced analytical methods requiring a lot of expertise and costly instruments are not always the most readily available methods to be applied in routine laboratories. Actions need to be taken at multiple levels to develop and validate fit-for-the-purpose analytical methods that can be easily applied by all stakeholders. Method validation assessment criteria need to be agreed upon and harmonised. Methods need to be developed that are specific to certain nanoparticle/matrix combinations and sometimes even for specific nanoparticle characteristics (e.g. surface properties). There are only few validated methods for nanoparticles in complex matrices and these have typically only been tested by a single laboratory.

The Surface Technology Group of the UK National Physical Laboratory (NPL) is also developing reference materials and documentary standards and is organizing inter-laboratory studies via the Versailles Project on Advanced Materials and Standards (VAMAS). The work focuses on surface chemical analysis, nanoparticle populations, graphene etc., which feeds into the projects of CEN and ISO. A VAMAS project on the measurement of the number concentration of nanoparticles involved 53 institutions mainly from Europe, including national metrology institutes such as the NPL, companies, research organisations and academia (Clifford, 2019).

Some major challenges in test methods and documentary standards for *in vitro* assays for medical drugs evaluation include that high-quality test methods are difficult, time-consuming and costly to develop, while even what is considered high quality may suffer from a variability of source data. There is also a critical shortage of pre-competitive test methods and documentary standards compared to the increasing number of drug product submissions containing nanomaterials. Moreover, the transferability of *in vitro* assays is also difficult and the number of successfully inter-laboratory tested methods is low (Nelson, 2019).

There are still a lot of deficiencies in inter-laboratory comparisons, and robust comparable results are in short supply. Concerning inter-laboratory studies, it can be noted that there are existing valued collaborations, for example European projects such as NANoREG (Gottardo et al., 2017) and NANoREG 2 (European Commission, 2020).

Lack of reference materials for different measurands (analytes) complicates this situation further. Table 4 lists those certified reference (nano)materials that are commercially available to-date. So far, certified reference nanomaterials provided by only a limited number of organisations such as the US NIST and the JRC exist mainly for particle size determination. There are no (certified) reference materials for nanoparticles in a matrix nor for micro- and nanoplastics. The challenges for method validation and the production of (certified) reference materials lie in the lack of harmonised analytical protocols, standardised methods and recognised templates for study data. The JRC also provides representative test nanomaterials through its Nanomaterials Repository (JRC, 2020). Notably, the JRC hosts a collection of tools and templates for generating and reporting – especially data relevant for hazard assessment – of nanomaterials, which derives from work within the NANoREG project (Jantunen et al., 2017; Totaro et al., 2017; Gottardo et al., 2019).

**Table 4:** *Commercially available certified reference (nano)materials*.

Name	Provider	Description	Certification
ERM-FD100	JRC	Colloidal silica in water (20 nm nominal)	Equivalent spherical diameter
ERM-FD101b	JRC	Colloidal silica (80 nm nominal)	Equivalent diameter
ERM-FD102	JRC	Colloidal silica (bimodal)	Equivalent diameter
ERM-FD103	JRC	Suspension of TiO_2_ nanorods	Minimum and maximum Feret diameter, maximum inscribed circle diameter, area-equivalent diameter, aspect ratio
ERM-FD304	JRC	Colloidal silica (40 nm nominal)	Equivalent spherical diameter
ERM-FD305/SRM 1992	JRC & NIST	Silica	electrophoretic mobility, zeta potential
ERM-FD306/SRM 1993	JRC & NIST	Silica	electrophoretic mobility, zeta potential
SRM 1898	NIST	Titanium Dioxide Nanomaterial	BET Specific Surface Area
SRM 1979	NIST	SRM 1979 - Powder Diffraction Line Profile Standard for Crystallite Size Analysis (Nano-Crystalline ZnO Powder)	Powder Diffraction Line Profile Standard for Crystallite Size Analysis
SRM 2483	NIST	Single-Wall Carbon Nanotubes (Raw Soot)	Mass fraction values
SRM 2484	NIST	Multiwall Carbon Nanotubes (Raw Soot)	Mass fraction values
SRM 1963a	NIST	Polystyrene Spheres (Nominal Diameter 100 nm)	Modal Sphere Diameter
SRM 1964	NIST	Polystyrene Spheres (Nominal Diameter 60 nm)	Modal Sphere Diameter
RM 8011, 8012, 8013	NIST	Gold (Nominal Diameter 10, 30, 60 nm)	NIST in-house validated
BAM P-109	BAM^a^	Activated nanoporous carbon	BET specific surface area
BAM P-116	BAM^a^	Titanium Dioxide (Anatase)	BET specific surface area

Note: CRMs available as of September 30, 2020. The following reference materials are also available from NIST without certificate: RM 8011 - Gold Nanoparticles (Nominal 10 nm Diameter), RM 8017 - Polyvinylpyrrolidone Coated Silver Nanoparticles (Nominal Diameter 75 nm); RM 8281 - Single-Wall Carbon Nanotubes (Dispersed, Three Length-Resolved Populations).

a BAM: German Federal Institute for Materials Research and Testing.

It is important to note that a single material can be certified reference material (CRM) for one property, but also a non-certified reference material (RM) for another property, and it can also be a representative test material (RTM) for a third property (Roebben et al., 2013). RMs and RTMs can play a crucial role in ensuring the reliability of toxicology assessment results. In the legislative context, measurement must be based on reliable methods (according to CEN and ISO standards, for example) with well-characterised materials (RTMs, RMs) used for method development and implementation. RMs and CRMs are needed for quality assurance to ensure that measurement results are comparable and correct such as for laboratory accreditation and method validation (Held, 2019). In support to the implementation of EU legislation including food and feed, food contact materials, chemicals, cosmetics, medicines and biocides, but also in support of research related to toxicology and eco-toxicology, the JRC develops and provides CRMs, RMs and RTMs in close collaboration with other prominent reference materials providers such as the US NIST (JRC, 2020b; NIST, 2020). The relevant and currently available certified nanomaterials are listed in Table 4. To respond to the urgent needs, the JRC is currently working on a number of nanomaterials (C)RMs embedded in complex matrices as well as on RMs for microplastics.

In addition to good quality and reproducible data, there should be greater clarity in terminology for standards. Overlapping standards from different organisations should be avoided. While there are a number of standardisation documents based on the use of specific instrument types and techniques, it may be more appropriate to develop standards on the properties of nanomaterials that are method-defined. Moreover, such standards should encompass ideally the entire method, starting with sample preparation, choice of instrument and reference materials. This would remedy the frequent need to use a combination of standards to obtain the desired result.

As already mentioned above, less is achieved to-date in the area of standards for micro-/nanoplastics in comparison to nanomaterials, which can explain why no clear regulatory requirements are existing yet. However, due to the increasing public concern, there is an urgent need for the development, validation and harmonisation/standardization of analytical approaches to understand the many challenges related to environmental contamination, human exposure and possible risks from small plastic particles.

## 8 Future directions

Since GSRS 2016, when the topic of nanotechnology was discussed as well, many new efforts have taken place towards harmonisation of guidelines and analytical approaches for quantification of nanomaterials and their risk assessment. This is due to the gradual increase in medical products that utilise nanomaterials together with a growing number of nanomedicines, but also due to the many other applications in the agri/food sector and other areas.

Although a lot of progress has been made, these topics warrant remained attention by regulatory scientists and regulators as well as research funders. Technological development is moving towards manipulability at the nanoscale, with developments in bionanotechnology representing only some examples. From a regulatory and policy perspective, many applications are at an early stage and not yet on the market, with the research in these early stages being technologically oriented, without much consideration on potential risks or safety or potential adverse effects. As the field develops, both risks and benefits should be taken into account, ideally as early as possible in the development process. A more holistic view on the benefits and potential risks, especially of complex nanomedicines is needed and can be achieved if scientific, regulatory communities and research funders work more closely together. There should be increased efforts to ensure that testing of nanomaterials and nanomedicines is rigorous, reproducible, and comparable between samples and situations, throughout the method and risk assessment development pipeline. Fit-for-the-purpose analytical tests must be further developed (and be complementary) to specific complex products. It is very much wished e.g. by pharmaceutical companies, that regulatory authorities get involved at the early stages of the process, not only when it comes to authorization (Pottier, 2019). Public-private partnerships are considered essential for the development of new medical solutions for patients.

For now, the applicable legal frameworks only start to consider the specificities of new materials at the nanoscale and their potential impacts. Safe-by-design could be an appropriate approach for such materials, starting early in the innovation process and balancing safety and functionality, taking into account the safety issues mentioned here. To facilitate the implementation of safe-by-design, key steps will include dialogue with stakeholders in a trusted environment. Vice versa, regulators will be informed in such a trusted environment about incoming innovations in good time and this will ensure regulatory preparedness. Altogether, this will contribute to the goal of appropriate nanotechnology governance, having faster, cheaper, effective, and safer products on the market.

There is a widespread concern deriving from the increase of plastic litter in the oceans and terrestrial environment that can affect ecosystems, biodiversity and potentially human health. This is making the issue of micro-/nanoplastics a multidisciplinary global issue. Research in this area has started only recently, and there are still many unresolved issues of terminology, definitions, sampling, characterisation and the assessment of hazard and exposure. As the term nanoplastics may be possible to be derived from the more generic term nanomaterials, it should be explored how far existing definitions and moreover methodological approaches used for nanomaterials can be applied for nanoplastics as well.

However, for both areas, nanotechnology/nanomaterials and nanoplastics, the main regulatory challenges are the lack of global standardisation of nomenclature, test methods or characterization, which can lead to divergence in regulatory approaches globally. This could be addressed through regulation, legislation, alignment and harmonisation, but no matter what the approach or combination of approaches are, the key success will be the involvement of all stakeholders (scientists, regulators, standardisation bodies, industry, patient representatives, consumers etc.) in creating a fit-for-purpose regulatory system.

The GCRSR will continue discussing these topics in their future meetings and summits. The JRC, as the European Commission's scientific in-house service and as a member of the GCRSR, offers open access to it is nanobiotechnology laboratory infrastructure to both academia and small medium enterprises (SMEs) (JRC, 2020) and is a partner in a number of European and international research projects, which can be used for investigations related to the characterization of nanomaterials.

To utilise evident synergies related to standardisation, an international working group should be established – ideally under the GCRSR, through a future GCRSR Standard Group – to monitor the development of guidance documents and standards worldwide and to identify priority areas and gaps that could be jointly addressed. The GCRSR could have a role as facilitator of communication among different standardisation bodies. This would include more coordination of laboratory-related activities such as inter-laboratory validation.

It should be stressed that nanoscience is still in the development phase thus well-established validated methods ready to be standardized are scarce to-date.

The authors invite therefore experts from all parts of the world to join such a group by contacting the authors of this paper.

## Disclaimer

The views expressed in this article are the personal views of the authors and may not be understood or quoted as being made on behalf of or reflecting the position of the agencies or organisations with which the authors are affiliated.

## Funding body information

The European Commission's 10.13039/501100000900Joint Research Centre has financially supported the organisation of the 2019 global summit and will also cover the costs for open access concerning the publication of this manuscript.

## CRediT authorship contribution statement

**Jacqueline Allan:** writing. **Susanne Belz:** writing, Data curation. **Arnd Hoeveler:** Supervision, reviewing. **Marta Hugas:** writing, reviewing. **Haruhiro Okuda:** reviewing. **Anil Patri:** Supervision, reviewing. **Hubert Rauscher:** writing, Data curation, reviewing. **Primal Silva:** reviewing. **William Slikker:** reviewing. **Birgit Sokull-Kluettgen:** reviewing. **Weida Tong:** Data curation. **Elke Anklam:** Supervision, writing, editing.

## Declaration of competing interest

The authors declare that they do not have known competing financial interests or personal relationships that could have appeared to influence the work reported in this paper.
